# Bulimia Symptoms in Russian Youth: Prevalence and Association With Internalizing Problems

**DOI:** 10.3389/fpsyt.2021.797388

**Published:** 2022-01-20

**Authors:** Roman A. Koposov, Andrew Stickley, Vladislav Ruchkin

**Affiliations:** ^1^Regional Centre for Child and Youth Mental Health and Child Welfare, Faculty of Health Sciences, UiT the Arctic University of Norway, Tromsø, Norway; ^2^Sechenov First Moscow State Medical University, Moscow, Russia; ^3^Department of Preventive Intervention for Psychiatric Disorders, National Institute of Mental Health, National Center of Neurology and Psychiatry, Kodaira, Japan; ^4^Stockholm Center for Health and Social Change, Södertörn University, Huddinge, Sweden; ^5^Child and Adolescent Psychiatry Unit, Department of Neuroscience, Uppsala University, Uppsala, Sweden; ^6^Child Study Center, Yale University Medical School, New Haven, CT, United States; ^7^Säter Forensic Psychiatric Clinic, Säter, Sweden

**Keywords:** bulimia symptoms, internalizing problems, prevalence, adolescents, gender

## Abstract

**Background:**

There has been limited research on bulimia symptoms in adolescents from the general population outside the United States. This study aimed to evaluate the prevalence of bulimia symptoms in Russian youth and explore the associations between a clinical level of self-reported probable bulimia nervosa (BN) and internalizing problems, binge drinking and functional impairment by gender.

**Methods:**

Data were collected from a representative sample of school students (N = 2,515, 59.5% female) from Northern Russia [age M (SD) = 14.89 ± 1.13 years]. Probable BN and internalizing psychopathology were assessed using self-report scales. Chi-square and independent sample *t*-tests were used to compare respondents' demographic characteristics and disordered eating behaviors. GLM multivariate analysis of covariance was used to assess the associations between probable BN, functional impairment and mental health problems (MHP) by gender.

**Results:**

Analyses showed that the 3-month prevalence of probable BN was higher in girls (3.9%) than in boys (1.2%). Probable BN was associated with depressive and anxiety symptoms, somatic anxiety, somatic complaints, binge drinking and functional impairment. Boys reported a higher level of problem scores in relation to probable BN.

**Conclusions:**

Our findings suggest that bulimia symptoms are prevalent in Russian adolescents and are associated with MHP and functional impairment. Timely recognition of bulimia symptoms and associated MHP is important for early prevention and intervention strategies.

## Introduction

Bulimia nervosa (BN) is an eating disorder (ED) characterized by multiple episodes of binge eating, followed by weight-related compensatory behaviors including self-initiated vomiting, laxative misuse, excessive exercise and fasting (1). Community-based studies have indicated that although body shape and weight concerns are common in adolescents, the prevalence of EDs nevertheless tends to be relatively low (2). The lifetime prevalence of BN among adolescents has been reported as ranging between 0.4 and 1.6% (3–5) with a mean onset age of 18 years old (6–8) and a significantly higher prevalence among females (9).

In the Russian Federation, information on disordered eating behaviors in adolescence is scarce. Stickley et al. (10) found that a large proportion of Russian adolescents expressed concern about their weight and body size, while an earlier study by Stevens et al. (11) showed that while Russian and American female adolescents were similar in terms of body size preference and the prevalence of dieting behavior, American girls were much more worried about being overweight. Other studies have reported higher levels of dissatisfaction with body weight among Russian, as compared to Finnish adolescent girls (12) and a higher drive for thinness among Russian female undergraduate students, when compared to their British counterparts (13).

Although BN is often considered to be primarily a female disorder, there has been a considerable increase in the attention being paid to males diagnosed with EDs (14–17). Most of these studies have focused on gender differences in the epidemiology and comorbidity of EDs. Studies have indicated that the prevalence of full or partial BN symptoms tends to be lower in male than female adolescents (18), with the male/female BN ratio ranging from 1:4 to 1:5 (5, 19). Adolescents with EDs can develop significant psychological and somatic problems and may have difficulties with school, family and social functioning (3, 16, 20–25). Previous studies have reported that severe purging behaviors are associated with different somatic problems such as dental, cardiovascular, gastrointestinal and other medical complications and might even be life threatening (26, 27). Various studies have also shown a clear association between binge eating and binge drinking (i.e., drinking a lot of alcohol in a short period of time) suggesting several overlapping aspects such as personality characteristics and affective features, and that both behaviors may have the same underlying purpose: to prevent or eliminate anxiety or other negative emotional states and may lead to similar negative consequences (28, 29). In adolescence, EDs are significantly more common in individuals with depressive or anxiety disorders (22). It has been hypothesized that depression and anxiety may co-occur with EDs as a result of neuroendocrinological disturbances brought on by weight loss (30). It is also possible that adversity in childhood might lead to affective and eating disorder psychopathology by lowering self-esteem and amplifying feelings of helplessness and body dissatisfaction (31, 32). Finally, it has also been proposed that episodes of binge eating may generate inwardly-directed negative emotions that may in turn, result in depressive and anxiety episodes (33).

As yet, however, it is unclear whether these mental health problems similarly co-occur with disordered eating in girls and boys. While a similar co-occurrence has been observed in some studies on binge eating (34, 35), gender differences have been reported in many others (36, 37). In particular, in girls binge eating has been associated solely with internalizing problems but among boys this behavior has been linked to internalizing and other problems including somatic complaints and binge drinking (16, 37, 38). In addition, although the body of research on the above-mentioned associations is growing quickly, until now, the vast majority of the research has been undertaken in Western countries and there has been little focus on EDs and associated mental health problems in the countries of Eastern Europe. This may be an important omission. Recent studies have highlighted that problematic eating attitudes and behaviors may be common in adolescents in Eastern Europe and in Russia (10, 39). Given this, further exploration of possible gender differences in bulimia symptoms and associated mental health problems in Russian adolescents is warranted with the goal of facilitating possible gender-oriented interventions prior to the onset of these behaviors.

Several previous studies have also reported that the manifestation of EDs and associated comorbid disorders among adolescents is impacted by age and socio-economic status (SES), with their prevalence increasing with age (40) and being higher in families with low incomes (41, 42).

Given that studies on the prevalence of BN symptoms and comorbid mental health problems (MHP) in adolescent males from the general population have been limited until now, and that there is still a scarcity of research on EDs/BN in Russia, the aims of the current study were: (1) to estimate the gender-specific prevalence of clinically significant bulimia symptoms (probable BN) in Russian youth and (2) to explore the associations between probable BN and internalizing problems, binge drinking and functional impairment, controlling for age and SES.

## Materials and Methods

### Sampling and Participants

The data used in this study came from a large epidemiological survey of students undertaken in the northern Russian city of Arkhangelsk. Details of the school survey and its methodology have been previously published elsewhere (43). A total of 2,847 reports were collected, but 332 students were excluded from the analytic sample because of missing values on one or more of the variables of interest. The youths in the excluded group reported lower levels of anxiety [*M* (*SD*) = 12.11 (5.60) vs. 13.39 (5.67), *t* = 3.40, *p* < .001]. Otherwise, the groups did not differ from each other on any other variables of interest. The final sample consisted of 2,515 students. Participants in the study sample ranged in age from 13 to 17 years [*M (SD*) = 14.89 (1.13)]. The composition of the sample was 59.5% female (*N* = 1,497), an accurate reflection of the local public school population. Boys, as compared to girls, were slightly younger [14.79 (1.11) vs. 14.96 (1.13), *t* = 3.73, *p* < 0.001], but did not differ in terms of SES [1.05 (1.14) vs. 1.00 (1.20), *t* = 1.12, *p* = 0.264]. Most of the participants (75.7%) came from two-parent families. According to the students' reports, 58% of their fathers and 60.1% of mothers had completed the equivalent of a high school education or beyond.

Ethical approval for the study was obtained from the Northern State Medical University in Arkhangelsk, Russia. It was performed in accordance with the ethical standards laid down in the 1964 Declaration of Helsinki and its subsequent amendments. The data were collected from a representative sample of students in the sixth to tenth grades in Arkhangelsk's general public schools (i.e., 10% of the students in each of the city's four districts). The participants were in classes that had been randomly selected from within schools that had themselves been randomly selected from a list of all schools in each of the city's districts. All of the students were invited to participate in the survey and complete the questionnaire during a regular school day. All information was collected anonymously. Each participant provided written informed consent before the questionnaire was administered, with both the parents (on behalf of their children) and the children themselves having the right to refuse to participate in the study. A total of 3.6% of the students or their parents declined to participate.

### Measures

*Disordered eating behaviors* were assessed using a shortened version of the Eating Disorder Diagnostic Scale (44). Specifically, it consists of four statements on the occurrence of anorexia and bulimia symptoms in the previous 3 months: “I worried a lot about how to stop gaining weight” (anorexia/bulimia), “I felt fat even when others told me I am too thin” (anorexia/bulimia), “I felt very upset about my overeating or weight gain” (anorexia/bulimia), and “I ate large amounts of food even when I didn't feel hungry” (bulimia). There were three response options: “Not true” (scored 0), “Somewhat true” (1), and “Certainly true” (2). In addition, two questions also assessed the frequency (per week) of compensatory behaviors to prevent weight gain, including (1) vomiting or using laxatives and (2) fasting (skipping at least 2 meals in a row) or engaging in excessive exercise, on a 5-point scale ranging from “0 times” (scored 0) to “More than 10 times” (4).

Positive responses (certainly true responses only) on the above items were used for coding DSM-5 criteria for bulimia (45), so that a proxy variable for *a clinical level of bulimia symptoms* (probable BN) was created. Diagnostic criterion A (recurrent episodes of binge eating) was coded based on the item: “I ate large amounts of food, even when I didn't feel hungry.” B and C criteria (recurrent inappropriate compensatory behaviors, such as using laxatives, vomiting and fasting or excessive exercise, that are intended to prevent weight gain at least once a week for 3 months) were assessed with two items: “About how many times per week have you made yourself vomit or used laxatives to prevent weight gain?” (at least once) OR “About how many times per week have you fasted (skipped at least two meals in a row) or engaged in excessive exercise to prevent weight gain?” (at least once). Criterion D (body shape and weight unduly influences self-evaluation) was coded based on a positive response for either of the following statements: “I felt fat even when others told me I am too thin,” OR “I felt very upset about my overeating or weight gain.” Positive symptom scores for all four diagnostic criteria were used to create a binary variable (0/1), which was used in all analyses and denoted as probable BN. This approach to assessing probable BN is in line with previous studies (39, 46).

*Depressive symptoms* were assessed with an adapted version of the Center for Epidemiologic Studies-Depression Scale (CES-D) (47). Research on adolescent populations has shown that both the CES-D (48) and its modified forms (49) have very good psychometric properties. The scale consists of 10 negative statements (e.g., “I felt really down”; “I have lost my interest in other people or things”), but an item on poor appetite was removed in order to avoid a potential overlap with eating problems. The presence of symptoms during the past month was assessed using a three-point answer scale [“Not true” (scored 0); “Somewhat true” (1); or “Certainly true” (2)]. The total scale score ran from 0 to 18 with higher scores indicating increased depressive symptomatology. Cronbach's α for this measure was 0.82. (All of the other alpha values reported below were calculated for the measures used in this study).

*Anxiety symptoms* were assessed with a scale (50) that consists of 12 items that inquire about worrisome or preoccupying thoughts and feelings (e.g., “I worry about other people liking me,” “I worry about doing something stupid or embarrassing”) with a three-point answer scale [“Not true” (scored 0); “Somewhat true” (1); or “Certainly true” (2)]. The total score ranged from 0 to 24 with higher scores indicating greater anxiety. Cronbach's α = 0.85.

*Posttraumatic stress* was evaluated with the Child Post-Traumatic Stress - Reaction Index (CPTS-RI) (51, 52). This 20-item scale measures the frequency of posttraumatic stress symptoms on a 5-point scale, ranging from “Never” (scored 0) to “Most of the time” (4). The total scale score ranged from 0 to 80, with higher scores representing increased levels of posttraumatic stress. Cronbach's α = 0.87.

*Somatic complaints* were assessed with a scale (50) that consists of 10 items that rate common somatic complaints in children and adolescents (53). The occurrence of past-month somatic symptoms (e.g., headache, stomach ache) was reported using a three-point scale [i.e., “Not true” (scored 0); “Somewhat true” (1); or “Certainly true” (2)]. The scale's total score ranged from 0 to 20 with higher scores indicating greater somatic symptomatology. Cronbach's α = 0.81.

*Somatic anxiety* was measured with a scale (54) that assesses somatic experiences (bodily or physiological processes, or sensations), which are frequently linked to anxiety in children and adolescents. The scale consists of 7 items (e.g., “I feel shaky,” “I feel butterflies in my stomach”) that were preceded by the introductory phrase (“Often when I worry…”). The response options were: “Not True (scored 0), Somewhat True (1), or Certainly True (2). The total score ranged from 0 to 14, with higher scores indicating higher levels of somatic anxiety. Cronbach's α = 0.81.

*Binge drinking* refers to the phenomenon of consuming a high volume of alcohol in a short period of time (55). It was assessed by asking students: “During the past 30 days, on how many days, if any, did you have 5 or more drinks of alcohol in a row, that is, within a couple of hours?” Responses were rated on a 5-point scale ranging from 0 days to 6 or more days, with the total score ranging from 0 to 4, where higher scores indicate increased binge drinking.

*Functional impairment* was assessed with a supplement of the Strengths and Difficulties Questionnaire (SDQ) (56, 57). The scale asks whether the respondent (1) thinks that he/she has difficulties in emotions, concentration, behavior, or in getting along with other people (with responses being given on a four-point scale ranging from “No” to “Yes, severe difficulties”) and (2) if so, inquires about the degree of distress (“How much do these problems upset or bother you?”); degree of functional impairment (“Do these difficulties interfere with your everyday life?”) in four areas: home life, friendships, classroom learning, and leisure activities; and degree of burden to others (“Do these difficulties make it harder for those around you?”). The items match DSM criteria, which stipulate that the symptoms of a disorder should result in significant distress or functional impairment. All items were scored as 0 for “Not at all” or “A little,” 1 for a “Medium amount,” and 2 for “A great deal.” The total score could range from 0 to 12, with higher scores indicating increased impairment. When the response was “No” to the first impact question, the whole impact score was set to zero. Cronbach's α = 73.

#### Proxy for Socio-economic Status (SES)

A proxy SES variable was created using students' reports on single family status (1/0), lower level of parental education (incomplete college education or lower, 1/0), and parental employment status [full time (0), part time (1), and unemployed (2)]. The total score ranged from 0 to 6, with higher scores indicating lower SES.

### Statistical Analyses

The Statistical Package for the Social Sciences (SPSS-25.0) was used to analyze the data. Univariate comparisons of the respondents' demographic characteristics and individual disordered eating behavior items were made with Chi-square and independent sample *t*-tests. The general linear models (GLM) multivariate analysis of covariance (MANCOVA) option was used to assess main and interaction effects across the fixed factors of probable BN (1/0) (as described earlier) and gender (boys = 1, girls = 0), while adjusting for age and SES covariates.

MANCOVA analyses were conducted with problem scores (depression and anxiety symptoms, somatic complaints, somatic anxiety, posttraumatic stress, binge drinking and functional impairment). Thus, we used a 2 (probable BN) X 2 (gender) design for assessing the differences in problem scores. The unique contribution of each of the two fixed factors, the two covariates, and one interaction term was assessed through follow-up between-subject tests and unstandardized parameter estimates derived from the MANCOVA. Results are presented as means (*M*) and standard deviations (*SD*), and for the individual outcomes of the between-subject tests as partial eta squared (η^2^), a common metric of effect size that represents the unique amount of variance explained by each predictor variable. Bonferroni corrections for multiple testing were applied (suggested *p* < 0.0166).

## Results

The prevalence of disordered eating behaviors by gender is presented in Table 1. The prevalence of all disordered eating behavior symptom types, except for vomiting or using laxatives, was significantly higher among girls as compared to boys. When comparing the other outcome variables by gender, boys (as compared to girls) reported lower scores on all of the problem scales, except for binge drinking (Table 2). Probable BN was also significantly more prevalent in girls than in boys, when using both a more stringent criterion (certainly true symptom ratings only) (3.9 vs. 1.2%, χ^2^ = 16.27, *p* < 0.001), and a broader criterion (somewhat true or certainly true symptom ratings) (13.4 vs. 3.5%, χ^2^ = 9.89, *p* < 0.001).

**Table 1:** Prevalence of different types of disordered eating behavior in the past 3 months by gender [*N* (%)].

**During the past 3 months**	**Boys**	**Girls**	**Chi-square^a^, *p*^b^**
I worried a lot about how to stop gaining weight	45 (4.4)	358 (23.9)	170.84; <0.001
I felt fat even when others told me I am too thin	38 (3.7)	276 (18.5)	119.48; <0.001
I ate large amounts of food even when I didn't feel hungry	88 (8.7)	185 (12.4)	6.66; <0.05
I felt very upset about my overeating or weight gain	40 (3.9)	254 (17.0)	99.96; <0.001
I made myself vomit or used laxatives to prevent weight gain (*at least once per week*)	34 (3.4)	36 (2.4)	2.07; ns
I fasted (skipped at least 2 meals in a row) or engaged in excessive exercise to prevent weight gain (*at least once per week*)	104 (10.5)	462 (31.2)	145.00; <0.001

*Prevalence described for “certainly true” responses, unless indicated otherwise*.

a *Chi-square, The Chi-square test*.

b *p, Significance value*.

*ns, Non-significant*.

**Table 2:** Problem scores [M (SD)] by bulimia symptoms in boys (B) and girls (G).

		**Probable BN^a^**			**Statistics for the gender**
					**comparisons in the total group**
		**Yes (*n* = 70)**	**No (*n* = 2,445)**	**Total group**	***t*, *p*-value**
Depressive symptoms	B	9.00 (2.52)	7.61 (2.90)	7.63 (2.89)	4.80, <0.001
	G	9.44 (2.10)	8.11 (2.64)	8.16 (2.64)	
Anxiety symptoms	B	18.42 (4.06)	12.27 (5.75)	12.34 (5.77)	7.68, <0.001
	G	16.00 (4.87)	14.02 (5.51)	14.09 (5.49)	
Somatic anxiety	B	6.33 (4.23)	3.49 (2.84)	3.53 (2.87)	11.85, <0.001
	G	5.67 (3.03)	4.90 (2.95)	4.93 (2.96)	
Somatic complaints	B	10.00 (4.94)	4.50 (3.49)	4.56 (3.56)	6.85, <0.001
	G	6.83 (4.20)	5.51 (3.60)	5.57 (3.63)	
Posttraumatic stress	B	32.17 (19.11)	17.52 (10.59)	17.70 (10.83)	8.15, <0.001
	G	28.43 (14.82)	21.11 (11.12)	21.39 (11.37)	
Binge drinking	B	0.75 (1.22)	0.87 (1.26)	0.86 (1.26)	0.71, >0.05
	G	1.17 (1.27)	0.82 (1.19)	0.83 (1.20)	
SDQ^b^ impact scale	B	1.42 (1.38)	0.68 (1.40)	0.68 (1.40)	4.08, <0.001
	G	1.84 (2.73)	0.91 (1.59)	0.94 (1.66)	

a *BN, Bulimia nervosa*.

b *SDQ, Strengths and Difficulties Questionnaire*.

When evaluating the differences in problem scores by probable BN (using a stringent criterion, i.e., certainly true symptom ratings only) {see Table 2 for descriptive statistics [*M (SD*)] by gender and Table 3 for the tests of between-subjects effects}, the main effect for the model was significant [Wilks' lambda = 0.927; *F*_(7, 2, 488)_ = 27.93, *p* < 0.001, η^2^ = 0.073]. With regard to specific effects, the main effect for Probable BN was significant [Wilks' lambda = 0.959; *F*_(7, 2, 488)_ = 7.35, *p* < 0.001, η^2^ = 0.042], with higher levels of all problem scores in those with Probable BN (Table 3). The main effect for Gender was also significant [Wilks' lambda = 0.992; *F*_(7, 2, 488)_ = 2.79, *p* = 0.007, η^2^ = 0.008]. The main effect for SES was significant [Wilks' lambda = 0.990; *F*_(7, 2, 488)_ = 3.64, *p* < 0.01, η^2^ = 0.010], suggesting higher levels of some problem scores (i.e., anxiety, somatic complaints and posttraumatic stress, see Table 3) in relation to lower SES. The main effect for Age was also significant [Wilks' lambda = 0.941; *F*_(7, 2, 488)_ = 22.29, *p* < 0.001, η^2^ = 0.059], indicating higher levels of problem scores with age. Regarding the interaction effect, Probable BN x Gender was significant [Wilks' lambda = 0.988; *F*_(7, 2, 488)_ = 4.46, *p* < 0.001, η^2^ = 0.012], suggesting that the differences in problem scores in relation to probable BN were gender-specific, with the follow-up tests showing differences in anxiety, somatic anxiety, somatic complaints and posttraumatic stress, with boys reporting higher levels of problem scores in relation to probable BN than girls.

**Table 3:** Effect sizes for each dependent variable (problems scores) (η^2^, *p*)^a^, ^b^.

	**Depressive**	**Anxiety**	**Somatic**	**Somatic**	**PTS^c^**	**Binge**	**SDQ^d^**
	**symptoms**	**symptoms**	**anxiety**	**complaints**		**drinking**	**impact scale**
Age	0.016, < .001	0.001, ns	0.000, ns	0.003, <0.01	0.002, <0.05	0.041, <0.001	0.006, <0.001
SES^e^	0.000, ns	0.002, *p* < 0.05	0.000, ns	0.004, <0.001	0.006, <0.001	0.000, ns	0.001, ns
Gender	0.001, ns	0.001, ns	0.003, *p* < 0.01	0.000, ns	0.000, ns	0.000, ns	0.002, *p* < 0.05
Probable BN^f^	0.006, <0.01	0.006, <0.001	0.014, <0.001	0.022, <0.001	0.030, <0.001	0.002, *p* < 0.05	0.009, <0.001
Probable BN × Gender	0.000, ns	0.002, <0.05	0.002, <0.05	0.005, <0.001	0.005, <0.001	0.000, ns	0.000, ns

a *η^2^, Eta squared*.

b *p, Significance value*.

c *PTS, Posttraumatic stress*.

d *SDQ, Strengths and Difficulties Questionnaire*.

e *SES, Socio-economic status*.

f *BN, Bulimia nervosa*.

*ns, Non-significant*.

As several outcomes were assessed at the same time in the same model, it is possible that outcome differences might have been concealed by simultaneous tests. To examine this possibility, a sensitivity analysis was performed where each outcome was assessed in a series of separate ANCOVAs. This produced results that were essentially the same as the findings from the MANCOVA.

Finally, as a large number of tests were undertaken, we also examined if the results would change when applying a (Bonferroni) correction for multiple testing. This showed that the interaction effects for probable BN × Gender only remained significant for somatic complaints and posttraumatic stress (suggested *p* < 0.0166).

## Discussion

The present study extended previous research by examining the gender-specific prevalence of clinically significant levels of bulimia symptoms in a large community-based sample of Russian adolescents and determining whether comorbid MHP associated with probable BN might be gender-specific. Our findings suggest that BN symptoms are common among Russian adolescents, are associated with a wide variety of problem behaviors, and that this association might be especially strong in boys.

The prevalence rates of probable BN in this study ranged from 1.2% in boys to 3.9% in girls. The finding of a higher prevalence of probable BN in girls is in line with previous research (3, 58, 59). While lower than in girls, the prevalence of probable BN in boys in the present study was still higher than in several earlier studies on BN (3, 5). It has been concluded that there is a lack of population-based research on EDs in adolescent males (60), while the previously reported female-to-male prevalence estimates have varied considerably from 10:1 (58, 59) to 3:1 (3). The present findings suggest that bulimia symptoms may be more prevalent in males than some previous studies have suggested, especially given that the prevalence of compensatory behaviors intended to decrease weight gain, such as induced vomiting or using laxatives, was similar by gender. Indeed, our finding of a similar prevalence of purging behaviors in boys and girls contrasts with several previous studies that have shown that such behaviors are more prevalent in females than in males (61–63). It is unclear what underlies the similarity in this study, but this intriguing finding deserves further investigation.

There was a strong association between probable BN and poorer mental health in this study. This finding accords with those from both clinical and epidemiological studies, which have shown that up to 88% of adolescents with BN may also suffer from another psychiatric disorder, and that many have two or more comorbid diagnoses (3, 22, 64), with particularly strong associations with mood and anxiety disorders (3). It has been suggested that anxiety might be central in both the etiology and maintenance of BN (65). In one psychometric evaluation of adolescents with EDs, almost 40% were diagnosed with anxiety, posttraumatic stress and obsessive-compulsive disorders (66). Our findings for binge drinking and functional impairment are also in line with those from earlier studies among adolescents, where significant associations were observed between probable BN and alcohol use [e.g., (24, 67, 68)] and between BN and a higher level of functional impairment [e.g., (69)]. Lewinsohn et al. (70) have previously shown that the presence of multiple, co-occurring MHP among adolescents with EDs has a significant impact on their functioning. Comorbidity also has important clinical implications, as there is some evidence that psychiatric comorbidity among those with EDs usually correlates with a more severe disease course and poorer prognosis for treatment (71).

Before applying an adjustment for multiple comparisons there were significant gender-specific differences in several problem scores in relation to probable BN, indicating that boys with probable BN may experience higher levels of psychiatric comorbidity than girls. Although it is unclear what underlies this finding it can be speculated that as EDs are much less common among boys, their onset might reflect the presence of an already existing high level of difficulty in psychological functioning that might be associated with a greater range of symptoms and high comorbidity. This result suggests that the detection and treatment of BN in boys is also an urgent task. However, boys are generally more reluctant to seek help for BN symptoms because of fears about experiencing rejection and feelings of shame, together with worries about confidentiality, which are among the factors that may have an impact on children's desire to seek EDs treatment in both genders (60). Moreover, reluctance to seek help may also be greater among males given the perceived stigma attached to, and cultural stereotypes surrounding EDs as exclusively female disorders (72, 73). Indeed, the effects of this might be especially pronounced for adolescent males in Russia, where traditional/masculine norms have been increasingly promoted in the transition years (74) and may have led to boys being even more reluctant to get treatment for EDs. Reducing the stigma surrounding EDs and improving detection of EDs/ED symptoms in young males is clearly a pressing task in clinical practice, particularly within pediatric clinical care.

## Limitations

We lacked information on some factors that would have helped us better elucidate probable BN, i.e., eating beliefs and attitudes, which have been identified as significant contributing factors in the development of EDs among adolescents from different countries (75). The data were cross-sectional and thus we were not able to determine the directionality of the observed associations, while self-reports may have been susceptible to different forms of bias. In particular, given the social stigma that is attached to eating problems (76), it is possible that socially desirable responding may have been an issue. Regarding directionality, it is uncertain whether MHP preceded the onset of BN symptoms or were a consequence of them (77). Determining the directionality of these associations may have important consequences for interventions aimed at preventing both BN and poorer mental health. Another limitation is that we were not able to examine these associations while also considering the sexual orientation of the participants. There is some evidence that bisexual and lesbian girls have higher rates of binge/purge behaviors, while data are less clear for boys (78). This is an important area for future research given that sexual minority adolescents are also more likely to experience poorer mental health (79). We had no information on the diagnostic history of the participants and were thus not able to determine if any of them had been diagnosed with or were receiving treatment for an ED. Finally, as several of the instruments used in this study have not been previously validated in Russia our findings should be considered exploratory.

## Conclusion

This study has shown that probable BN is prevalent in Russian adolescents and is associated with worse mental health and functional impairment. Our findings highlight the necessity of detecting eating problems in boys and girls as early as possible, and indicate that once detected, treatment plans should include comprehensive screening for a range of mental health disorders, while interventions should be able to potentially address multiple diagnoses among adolescents. The results of this study also suggest that it might be potentially beneficial to screen for eating problems in children with MHP—especially boys.

## Data Availability Statement

The raw data supporting the conclusions of this article will be made available by the authors, without undue reservation.

## Ethics Statement

This study involving human participants was approved by the Ethical Committee at the Northern State Medical University in Arkhangelsk, Russia. Written informed consent to participate in this study was provided by the participants' legal guardian/next of kin.

## Author Contributions

VR, RK, and AS were involved in the conceptualization and design of the study. RK and VR conducted the data collection and drafted the manuscript. VR conducted the data analysis. All authors contributed to the article and approved the submitted version.

## Conflict of Interest

The authors declare that the research was conducted in the absence of any commercial or financial relationships that could be construed as a potential conflict of interest.

## Publisher's Note

All claims expressed in this article are solely those of the authors and do not necessarily represent those of their affiliated organizations, or those of the publisher, the editors and the reviewers. Any product that may be evaluated in this article, or claim that may be made by its manufacturer, is not guaranteed or endorsed by the publisher.
